# Randomised controlled trial of perinatal vitamin D supplementation to prevent early-onset acute respiratory infections among Australian First Nations children: the ‘D-Kids’ study protocol

**DOI:** 10.1136/bmjresp-2023-001646

**Published:** 2023-08-16

**Authors:** Michael J Binks, Amy S Bleakley, Susan J Pizzutto, Michelle Lamberth, Verity Powell, Jane Nelson, Adrienne Kirby, Peter S Morris, David Simon, E Kim Mulholland, Geetha Rathnayake, Amanda J Leach, Heather D'Antoine, Paul V Licciardi, Tom Snelling, Anne B Chang

**Affiliations:** 1 Child Health Division, Menzies School of Health Research, Charles Darwin University, Casuarina, Northern Territory, Australia; 2 Department of Obstetrics and Gynaecology, Royal Darwin Hospital, Tiwi, Northern Territory, Australia; 3 National Health and Medical Research Council Clinical Trials Centre, University of Sydney CAR, Glebe, New South Wales, Australia; 4 Department of Paediatrics, Royal Darwin Hospital, Tiwi, Northern Territory, Australia; 5 New Vaccines Research Group, Murdoch Children's Research Institute, Parkville, Victoria, Australia; 6 Epidemiology and Public Health, London School of Hygiene & Tropical Medicine, London, UK; 7 Virtus Diagnostics, Sydney, New South Wales, Australia; 8 Department of Paediatrics, University of Melbourne VCCC, Parkville, Victoria, Australia; 9 School of Public Health, The University of Sydney, Sydney, New South Wales, Australia; 10 Wesfarmers Centre of Vaccines and Infectious Diseases, Telethon Kids Institute, Nedlands, Western Australia, Australia; 11 Centre for Children's Health Research, Queensland University of Technology, Brisbane, Queensland, Australia

**Keywords:** Respiratory Infection, Bacterial Infection, Viral infection, Pneumonia, Infection Control, Innate Immunity

## Abstract

**Introduction:**

Globally, acute respiratory infections (ARIs) are a leading cause of childhood morbidity and mortality. While ARI-related mortality is low in Australia, First Nations infants are hospitalised with ARIs up to nine times more often than their non-First Nations counterparts. The gap is widest in the Northern Territory (NT) where rates of both acute and chronic respiratory infection are among the highest reported in the world. Vitamin D deficiency is common among NT First Nations neonates and associated with an increased risk of ARI hospitalisation. We hypothesise that perinatal vitamin D supplementation will reduce the risk of ARI in the first year of life.

**Methods and analysis:**

‘D-Kids’ is a parallel (1:1), double-blind (allocation concealed), randomised placebo-controlled trial conducted among NT First Nations mother–infant pairs. Pregnant women and their babies (n=314) receive either vitamin D or placebo. Women receive 14 000 IU/week or placebo from 28 to 34 weeks gestation until birth and babies receive 4200 IU/week or placebo from birth until age 4 months. The primary outcome is the incidence of ARI episodes receiving medical attention in the first year of life. Secondary outcomes include circulating vitamin D level and nasal pathogen prevalence. Tertiary outcomes include infant immune cell phenotypes and challenge responses. Blood, nasal swabs, breast milk and saliva are collected longitudinally across four study visits: enrolment, birth, infant age 4 and 12 months. The sample size provides 90% power to detect a 27.5% relative reduction in new ARI episodes between groups.

**Ethics and dissemination:**

This trial is approved by the NT Human Research Ethics Committee (2018-3160). Study outcomes will be disseminated to participant families, communities, local policy-makers, the broader research and clinical community via written and oral reports, education workshops, peer-reviewed journals, national and international conferences.

**Trial registration number:**

ACTRN12618001174279.

**WHAT IS ALREADY KNOWN ON THIS TOPIC**

- Vitamin D deficiency (<50 nmol/L) is common among Australian First Nations infants at birth and linked to an increased risk of acute respiratory infection (ARI) hospitalisation in the first year of life. The most recent systematic review of clinical trials suggests that vitamin D supplementation could significantly reduce the risk of childhood ARI.

**WHAT THIS STUDY ADDS**

- ‘D-Kids’ is the first randomised controlled trial (RCT) to evaluate vitamin D supplementation as a novel strategy for reducing early-onset ARIs among Australian First Nations infants. This study extends the best local and international evidence, implementing a practical weekly vitamin D dose over known high-risk period of both vitamin D deficiency and ARI. Importantly, this RCT will monitor vitamin D levels through pregnancy and infancy and determine whether supplementation impacts the paediatric immune response and the acquisition of respiratory pathogens.

**HOW THIS STUDY MIGHT AFFECT RESEARCH, PRACTICE OR POLICY**

- This trial will validate the utility of vitamin D against infant ARI, guide best practice for supplementation and provide much needed local evidence on vitamin D reference ranges in pregnancy and infancy.

## Introduction

### Background and rationale

Acute respiratory infections (ARIs) remain the greatest global cause of childhood morbidity and mortality.^1^ The largest burden occurs among socioeconomically disadvantaged populations.^1^ In Australia, First Nations children are hospitalised with ARIs up to nine times more often than other children.^2^ The gap is widest in the Northern Territory (NT) where, despite government funded healthcare, high vaccine coverage and almost universal early breast feeding,^3 4^ rates of early-onset pneumonia (20% hospitalised in first year),^4^ otitis media (OM) (90% at age 6 months)^5^ and chronic suppurative lung disease (1 in 68 in Central Australia)^6 7^ are among the highest reported in the world. Further, the burden of ARI hospitalisation in the NT has changed little over the last two decades.^3 8^ Slow progress in improving health services and addressing the social determinants of health drives the need for novel, effective, community endorsed, evidence-based interventions to reduce the burden of ARIs in the region. Oral supplementation of vitamin D, integral to immune function,^9 10^ is one such intervention that could reduce infants’ susceptibility to ARIs.^11 12^

Vitamin D is a steroid-like molecule generated predominantly on exposure of skin to sunlight with the remainder coming from the diet.^13^ Cutaneously generated (vitamin D_3_) or ingested vitamin D (vitamin D_2_ or D_3_) is hydroxylated by the liver into 25-hydroxy-vitamin D (25OHD) which is considered the best measure of vitamin D status. The active hormonal form of vitamin D, 1,25 hydroxy-vitamin D3 (1,25OH_2_D_3_) is produced by the kidney or locally by specialised cells of other systems as required. Both 25OHD and 1,25OH_2_D_3_ are transported in the circulation by the vitamin D binding protein.^14^ Active vitamin D regulates gene expression via the vitamin D receptor (VDR).^15^ Conventionally, vitamin D is known for its role in regulating calcium metabolism though the effects of vitamin D and its metabolites are much broader.^16^

Most immune cells express both the VDR and the enzymes (1α-hydroxylase) necessary to locally convert circulating 25OHD into active 1,25OH_2_D_3_. As such, immune cell responses are affected by the availability of circulating 25OHD. On the activation of innate immune cells, 1,25OH_2_D_3_ is involved in the modulation of over 200 human genes (~1% of the total)^17^ including those involved in pathogen sensing and clearance (eg, ↑ cathelicidin), and control of inflammatory responses (eg, ↓IFNγ, ↑IL10).^9^ Vitamin D also influences adaptive immunity by modulating dendritic cell (DC) and T cell phenotypes, promoting a tolerogenic T-helper 2 response and inducing the expansion of regulatory T cells.^18^ The net effects of vitamin D against infection appear to be simultaneous promotion of antimicrobial activity, and control of excessive inflammatory and adaptive immune responses.

Vitamin D deficiency (<50 nmol/L) is prevalent in many populations globally and has been repeatedly linked to an increased risk of childhood ARI.^19–23^ Importantly, pregnancy is a period of increased demand for vitamin D. Levels tend to decline towards term^24–27^ and breast milk offers a relatively poor source of vitamin D.^28^ As such, vitamin D deficiency is common at birth and it can take up to 6 months for infants to achieve sufficiency.^24 27^ Our prospective cohort study in northern Australia demonstrated that mean cord blood 25OHD levels were 48% lower than maternal 25OHD levels at 32-week gestation, resulting in a high rate of neonatal deficiency (44% <50 nmol/L).^24^ Importantly, this study found that mean cord blood vitamin D was significantly lower among infants who were subsequently hospitalised with an ARI in the first year of life than in those who were not (37 nmol/L v 56 nmol/L; p=0.025).^24^

To address deficiency in pregnancy, doses of 2000–4000 IU/day from 12 to 16 weeks gestation have been shown to be safe and effective.^29^ For infants with deficiency, supplementation with 400 IU (for levels between 30 and 50 nmol/L) to 1000 IU (for <30 nmol/L) per day for 3 months is recommended depending on the severity of deficiency. Infant doses up to 1000 IU/day^27^ and bolus doses of 100 000 IU^30^ have been used safely. It remains unclear whether vitamin D supplementation in pregnancy and infancy can reduce the burden of early-onset infant ARIs.

A 2017 meta-analysis of individual-level data from 10 933 participants in 25 randomised controlled trials (RCTs)^12^ and subsequent 2021 update of aggregate data over 48 000 participants in 46 RCTs^31^ found that vitamin D supplementation reduced the odds of experiencing at least one ARI episode by 12% (OR 0.88 (95% CI 0.81 to 0.96))^12^ and 8% (OR 0.92 (95% CI 0.86 to 0.99)),^31^ respectively, compared with placebo. This reduction was found despite considerable population (ethnicity, age, socioeconomic status, baseline vitamin D) and design (vitamin D dosage/duration and outcome measure) heterogeneity. Importantly, the data available at the commencement of our study suggested both daily and weekly vitamin D dosing were effective against ARI (OR 0.81 (95% CI 0.72 to 0.91)).^12^ More recently, the largest effects have been seen in those receiving daily supplementation of ≥400 IU (OR 0.70 (95% CI 0.55 to 0.89)).^31^ In general, children 1–15 years of age appear to receive the most benefit (OR 0.71 (95% CI 0.57 to 0.93)) and large bolus doses at monthly or greater intervals have been ineffective.^12 31^ A New Zealand RCT found that compared with placebo controls, perinatal vitamin D supplementation (mothers from 27-week gestation to birth, infants from birth to age 6 months) maintained infant vitamin D status >50 nmol/L at birth and in infancy^27^ and significantly reduced ARI primary care presentations among infants (relative risk reduction of 12% (≥1 ARI, 99% vs 87%,) and incidence rate reduction of 37.5% (4.0 vs 2.5 ARI/child/year)) in the 12-month postsupplementation (infant age 6–18 months).^11^ Overall, few of the reviewed RCTs dosed weekly (6/46) or during the perinatal or neonatal period (4/46)^12 31^ where deficiency is common.^24 26 27^ As far as we are aware, none have done both.

### Objectives

The primary aim of our ‘D-Kids’ RCT is to determine whether practical weekly vitamin D supplementation (compared with placebo) of mothers (from 28^+0^ to 34^+6^ weeks gestation until birth) and their infants (from birth until age 4 months) reduces the incidence of ARI (hospital or primary care presentations) among high-risk Australian First Nations infants during their first 12 months of life.

The secondary aims are to determine whether the supplementation strategy above: (A) reduces vitamin D deficiency at birth and infancy; (B) enhances neonatal immune responses and (C) reduces the prevalence of nasal respiratory pathogens in infancy.

### Hypotheses

We hypothesise that perinatal vitamin D supplementation will reduce the incidence of ARIs (vs placebo); maintain vitamin D levels >50 nmol/L at birth and throughout infancy; promote optimal immune responses to pathogen challenge; and reduce the frequency of pathogens detected in the nose.

## Methods

### Trial design and setting

**Figure 1:**
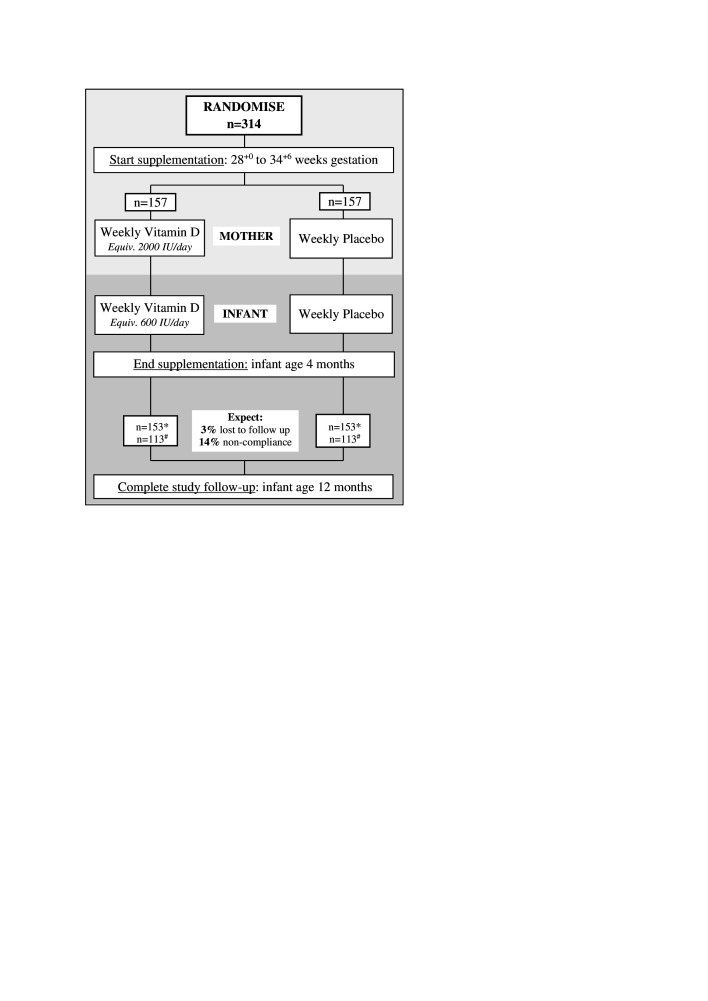
The ‘D-Kids’ randomised controlled trial, flow diagram. (1 IU=0.025 µg or 25 ng). Evaluable sample size: *Intention to treat; ^#^Per protocol. IU, international unit.

‘D-Kids’ is a parallel (1:1), double-blind, allocation concealed, randomised, placebo-RCT of weekly perinatal vitamin D supplementation (figure 1). The trial is being conducted among First Nations families in urban and remote communities of Australia’s NT. The NT is sparsely populated with approximately 229 000 residents spread across 1.4 million km² (0.16 people per km²).^32^ Approximately 70% of NT First Nations families reside in remote communities.^32^ Recruitment started in February 2019 and the study is scheduled for completion by the end of 2024.

### Patient and public involvement

The ‘D-Kids’ trial was built on respectful engagement and longstanding relationships with Australian First Nations families, hospitals and healthcare centres. Prior to study conduct, we sought the views of First Nations community councils and parents regarding the trial intervention and design acceptability. Our small prestudy survey of local families indicated a willingness to participate in such a study (24/25, 96%). Formal approval for this RCT was received from all communities involved and we have implemented a First Nations Reference Group model of governance. Importantly, the study team includes First Nations investigators, clinicians, health practitioners and trainees.

### Eligibility criteria

#### Inclusion criteria

Pregnant First Nations women with a current gestation of 28^+0^ to 34^+6^ weeks, aged 17–40 years (inclusive) residing in a participating community and intending to do so until their infant reaches 12 months of age. Eligibility includes dichorionic diamniotic (DCDA) twin pregnancies and previously enrolled mothers.

#### Exclusion criteria

Enrolment in other research that could influence the outcomes of this study, monochorionic diamniotic twin pregnancies, current use of prescribed vitamin D in pregnancy >400 IU/day (or equivalent), current self-supplementation with vitamin D >400 IU/day, current use of illicit or cytotoxic drugs (excluding marijuana and alcohol), antenatal hypercalcaemia in this pregnancy (serum calcium >2.8 mmol/L or a urinary calcium:creatinine ratio >1 on two occasions), uncontrolled thyroid disorders, chronic kidney disease (≥stage 4), known anaphylactic allergy or a history of or current kidney/bladder stones.

#### Additional criteria

Obstetrician approval is necessary for mothers with a history of >2 preterm births at <34 weeks gestation, DCDA twin and other high-risk pregnancies. Paediatrician approval is sought for ongoing infant study participation if born <36 weeks, admitted to the intensive or special care units, discharged on vitamin D 400 IU/day (Pentavite) or found to have a congenital anomaly. Babies born <36 weeks gestation and entering the special or intensive care nursery will not receive any study medicine until cleared to do so by the special care clinical team.

### Intervention

Eligible mother–infant pairs are randomised with equal probability to receive either weekly vitamin D (coconut oil plus cholecalciferol) or placebo (coconut oil only).

Both liquid active and placebo study medicines are manufactured and supplied by Ddrops, Ontario, Canada. Ddrops recommends storage in an upright position between 5°C and 30°C though the product has passed long term stability tests at 40°C. Stock medicine bottles and prefilled syringe doses (capped and placed in a labelled envelope) were both stored in the Menzies School of Health Research (MSHR) pharmacy at ambient temperature (approximately 21°C) prior to dosing. Mothers who received doses for self-administration were instructed to keep them in a secure, unrefrigerated location inside their house.

The maternal vitamin D dose is 14 000 IU/week (equivalent to 2000 IU/day); the infant dose is 4200 IU/week (equivalent to 600 IU/day). Mothers vitamin D or placebo commences at enrolment (28^+0^ to 34^+6^ weeks gestation) and continues until delivery (table 1, figure 1). Infant vitamin D or placebo commences at birth and continues until 4 months of age. Infants discharged from hospital with a recommendation to take oral vitamin D (400 IU/day) receive a reduced vitamin D dose of 3000 IU/week (equivalent to 430 IU/day).

**Table 1:** Participant timeline

Study visit:	0	1	2	3	4
Gestation (weeks)/infant age (months)*	20^+0^–27^+6^	28^+0^–34^+6^	0	4	12
Enrolment:					
Eligibility screen	✓	✓			
Informed consent/random allocation		✓			
Intervention:					
Mother (vitamin D or placebo)		✓	✓		
Infant (vitamin D or placebo)			✓	✓	
Primary outcome:					
Incidence of medically attended ARI episodes†			✓	✓	✓
Secondary outcomes:					
Clinical					
Prevalence of ≥1 medically attended ARI episode			✓	✓	✓
Time to first medically attended ARI episode			✓	✓	✓
Incidence of (1) pneumonia, (2) bronchiolitis, (3) otitis media			✓	✓	✓
Biological					
Vitamin D levels (circulating 25OHD_3_ concentration)		✓	✓	✓	✓
Systemic immunology (phenotype, CBMC challenge, vaccine Ig’s)			✓	✓	
Nasal microbiology (bacterial and viral pathogen prevalence)				✓	✓

*Window for visit 2 or birth visit: 0–14 days; visit 3 or 4-month visit: 3.5–6 months; visit 4 or 12-month visit: 11–18 months.

†Hospital or primary healthcare presentation.

ARI, any acute respiratory infection; CBMC, cord blood mononuclear cells; 25OHD, 25-hydroxy-vitamin D.

Study medicines are self-administered orally via prefilled syringes (mother: 0.4 mL; infant 0.2 mL). though study staff assist as requested. Doses are ideally taken 7 days apart. A dose delayed more than 11 days is considered a missed dose and the next dose taken as scheduled. There is no catch up for missed doses and >3 consecutive missed doses is considered a protocol deviation.

#### Adherence

Participant retention and adherence to the protocol are facilitated through regular contact via telephone, short message service, clinic or home visits. Contact is weekly during dosing (starting 7 days from previous dose) then monthly thereafter until the final study visit. A maximum of three consecutive days of contact are attempted for each scheduled visit or dose. Where possible, ingestion of study medicine is supervised by clinic, hospital or study staff. Unsupervised, self-administered doses are verified by phone. When dosing is complete the remaining volume of the used bottle is recorded, and it is stored securely and separately from the unused medicines. Empty dosing syringes are not collected.

#### Relevant concomitant care

‘D-Kids’ is a pragmatic trial. Outside the eligibility criteria all concomitant care and interventions will be allowed unless a specific contraindication to vitamin D therapy arises and discontinuation is recommended.

#### Criteria for discontinuing

Participants can withdraw from the study at any time: either entirely (no further medication, visits or collection of information) or partially (withdraw from medication and/or study visits). Partial withdrawals continue to contribute passive follow-up data (ie, medical record reviews in line with the primary study outcomes).

### Outcomes

#### Primary outcome

The primary outcome is the incidence of ARI episodes receiving medical attention in the first 12 months of the infant’s life. ARI episodes are identified via electronic medical records using established methods.^33–35^ Our ARI case definition encompasses any episode of hospital or primary healthcare documented in the medical records as either an acute lower respiratory infection, acute upper respiratory infection or OM. Associated signs, symptoms and observations are comprehensively documented for each ARI episode. Clinical case definitions and diagnostic algorithms are described in detail in online supplemental material 1. ARI episodes are considered unique if separated by ≥14 days. For hospitalisations, this gap begins at the previous discharge date.

10.1136/bmjresp-2023-001646.supp1 Supplementary data

#### Secondary outcomes

Pneumonia, bronchiolitis and OM (incidence) will be analysed as specific ARI subgroups (online supplemental material 1). Hospitalisation, antibiotic and oxygen therapy will be used as measures of severity.

Circulating 25OHD concentrations are measured in maternal blood at baseline (≤34^+6^ weeks) and at birth, in cord blood, and in infant blood at birth, 4 and 12 months, using high-performance liquid chromatography (HPLC) as described previously.^36 37^ Blood samples for vitamin D analysis include both plasma and dried blood spots (DBS). DBS-based 25OHD concentrations will be adjusted for haematocrit as necessary.^38^ Vitamin D levels <50 nmol/L are considered deficient.

The prevalence of nasal pathogens will be determined via nasal swabs, collected from infants at ages 4 and 12 months. Swabs will be screened for key respiratory bacteria (*Streptococcus pneumoniae*, non-typeable *Haemophilus influenzae* (NTHi)) and viruses (RSV) using WHO culture^39^ and standardised RT-PCR methods, respectively.^40 41^

#### Tertiary outcomes

Immune function will be assessed through analysis of whole blood, isolated cord blood mononuclear cells (CBMCs),^42^ plasma, breast milk and saliva samples. Immune cell populations will be characterised at birth (cord blood, CBMCs) and infant age 4 months (venous blood) using flow cytometry.^43^ CBMC-mediated immune responses to in vitro pathogen challenge will be assessed as previously described.^42^ Immune markers will be measured directly in infant plasma and saliva samples at birth and age 4 months using ELISA.^42^ Maternal pertussis vaccine induced antibodies will be measured in cord blood plasma and breast milk collected at birth using ELISA.^44^ Infant pneumococcal conjugate vaccine (PCV) induced antibodies will be measured in saliva and blood samples at birth and 4 months using ELISA.^45^ Given the explanatory and mechanistic nature, immunological outcomes will be presented separately to the main outcomes.

#### Future outcomes

Where volumes allow, blood aliquots will be stored in RNA preservative (RNA later, Invitrogen) for further characterisation of immune effector genes. Circulating calcium levels (via routine pathology services), breast milk vitamin D levels (via an adapted HPLC method)^38^ and polymorphisms in key vitamin D pathway genes^46^ (eg, the vitamin D receptor gene, VDR) will also be evaluated. Stool samples are also collected (OMNIgene, DNA Genotek) from a subset of participants for gut microbiome analysis.^47 48^ Additional funding has been secured (NHMRC 2014930) for follow-up of infant lung function to age 6 years.

### Recruitment strategies

‘D-Kids’ partners with local NT hospitals, community healthcare and pathology services. These services facilitate recruitment (and follow-up) through notification of pregnant women receiving care at their site. Once identified the study team engage potential participants at antenatal care visits.

### Study visits

Enrolment, study intervention, follow-up (table 1) and biological sampling (table 2) are achieved through four main study visits.

**Table 2:** Sample collection summary

Study visit:	1		2		3		4	
Gestation (weeks)/infant age (months)*	28^+0^–34^+6^		0		4		12	
**Samples**	**Mother**	**Infant**	**Mother**	**Infant**	**Mother**	**Infant**	**Mother**	**Infant**
Venous blood	✓		✓	✓		✓		✓
Cord blood				✓				
Breast milk			✓					
Saliva				✓		✓		
Nasal swab						✓		✓

*Window for visit 2, or birth visit: 0–14 days; visit 3 or 4-month visit: 3.5–6 months; visit 4 or 12-month visit: 11–18 months. Venous blood is collected by heel or finger prick onto Guthrie cards. Cord blood collected by midwives is split into three ways: spotted onto a Guthrie card; into a paediatric heparinised blood tube (for plasma) and into tubes containing media to be used for immunology studies. An aliquot of cord blood in media is used immediately for immune phenotyping then cord blood mononuclear cells are isolated from the remainder and stored within 24 hours for future batched challenge assays. Cord and venous plasma are stored for batched measurement of vitamin D levels (where volume allows), immune markers, vaccine antibodies. Blood spots (Guthrie cards) are used exclusively for measurement of vitamin D levels. Breast milk (collected at least 3 days post partum to avoid colostrum), saliva and nasal swabs (collected into skim milk, tryptone, glucose and glycerol media) are stored immediately until required for outcome analysis. All samples (apart from blood spots) are stored at −80°C.

Visit 0, screening: Families are approached during antenatal appointments (20^+0^ to 34^+6^ weeks gestation) at participating hospitals and community healthcare clinics and screened for interest and basic eligibility (maternal and gestational age, ethnicity, community of residence).

Visit 1, enrolment: Pregnant mothers are eligible for enrolment at 28^+0^ to 34^+6^ weeks gestation. At this visit, we provide a detailed explanation of the study rationale and requirements using plain language study material (including a participant information sheet, pictorial flipchart and consent form). First Nations team members translate materials as necessary. Interested families are formally invited to participate in the study. With written informed consent and confirmation of eligibility, mothers are enrolled and randomly allocated to the intervention or placebo arm. A baseline blood sample is collected prior to supervised administration of the first dose of study medicine.

Visit 2, birth: At delivery, cord blood is collected by the attending obstetricians or midwives and transported to the laboratory for processing within 24 hours. Study staff visit mothers and their babies within 14 days of birth. The visit coincides with the end of the maternal supplementation. Blood and breast milk (at least 3 days post partum) are collected from mothers. Blood and saliva are collected from neonates. Infant study medicine is commenced, and the first dose is administered.

Visit 3, infant age 4 months: Families are visited when infants are 3.5–6 months of age, coinciding with the end of the infant supplementation period (16th week post partum). An infant blood, nasal swab and saliva sample are collected.

Visit 4, infant age 12 months: The final study visit occurs between infant ages 11–18 months. An infant blood and nasal swab are collected. Families receive a small thankyou gift for their contribution.

Routine data are collected at each visit, including personal, household and community demographics, medical and vaccination history, smoke exposure, time spent indoors and use of vitamin D or other supplements, breastfeeding status and infant growth metrics. Serious adverse events (SAEs) and clinical outcomes are monitored via maternal and infant medical records (hospital and primary care) throughout follow-up.

### Sample size

Our sample size of 314 (figure 1) is expected to provide 90% ‘intention-to-treat’ and 80% ‘per-protocol’ study power to detect a 27.5% relative reduction^11^ in new ARI episodes between those receiving vitamin D supplementation (2.54/year) compared with placebo (3.50/year). Power calculations are based on local ARI rates,^3 8 49^ 14% non-compliance, 3% lost to follow-up and assume the outcome data fit a negative binomial distribution (dispersion factor, k=0.4).^50^ Our RCT’s predicted effect size is consistent with a similar New Zealand study by Grant *et al*.^11^ We expect a low drop-out rate because the primary outcome is informed by passively collected medical record data for each child.

### Assignment of interventions

#### Sequence generation

Sequential randomisation codes were computer-generated using permuted blocks (two blocks of differing sizes), stratified by community (urban and remote). The allocation ratio within these strata is 1:1 (vitamin D: placebo). There is one random allocation per mother–infant pair.

#### Allocation concealment

An independent study statistician generated the randomisation codes. Medication bottles are labelled by an independent clinical pharmacist from Royal Darwin Hospital. Labels incorporate a clear specification for mother or infant to avoid mix-up. Sequentially labelled mother and infant bottle pairs are stored in strata-specific boxes at room temperature in a secure facility. Active and placebo study medicine bottles and administration syringes are identical. Liquid medicines are indistinguishable in appearance and taste.

#### Allocation implementation

Good Clinical Practice (GCP) trained research nurses or First Nations health practitioners allocate the study medicine to each mother–infant pair by selecting the next sequentially labelled (prerandomised) study medication from the appropriate stratification group. The allocation sequence number is recorded by the research team on the data collection form (DCF), the database and in the participant’s medical record. Infants receive the same allocation as their mother.

#### Blinding

The study is double-blinded. All investigators, participants, carers, hospital and clinic staff are blinded to the treatment group until completion of study follow-up. Unblinding is permissible if the independent data safety monitoring committee (iDSMC), principal investigator or appropriate qualified delegate is compelled by evidence of a safety concern.

### Data collection, management and analysis

#### Data collection methods

All study visit and clinical data are recorded by research nurses on standardised paper-based DCFs. Protocol adherence (deviations/violations) are continuously monitored and documented by study staff. Established methods are used to document episodes of respiratory infection from medical records.^33–35^ Collection and processing times, and quality measures are recorded for all biological samples. A GCP compliant protocol is employed if corrections are made to paper-based records. All study documents are securely retained at MSHR.

#### Sample collection

Established methods are being used for the collection and processing of blood, breast milk, nasal, stool and saliva samples.^4 34 35 42 51^ Briefly, whole cord blood is collected via venepuncture: (1) into three (10 mL) heparinised tubes containing an equal volume of RMPI (Sigma-Aldrich) cell culture media; (2) into heparinised paediatric blood tubes (<1 mL) and (3) onto Guthrie cards (~80 µL). Maternal and infant venous bloods (finger or heel prick) are collected first onto Guthrie cards (filling designated spot to the edge) and then into heparinised tubes if the volume is sufficient. The cord blood samples collected in media are kept at room temperature and an aliquot is used for immunophenotyping^43^ prior to isolation of CBMCs. Plasma is obtained from all other heparinised blood samples following centrifugation. Guthrie card samples are dried for 24 hours then stored as DBS in an envelope at room temperature. Nasal samples are collected with a sterile wire shafted swab (Copan) then placed into skim milk-tryptone-glucose-glycerol broth. All samples (except cord blood-media samples and DBS) are kept at 4°C during processing. Isolated CBMCs and samples (except DBS) are stored in cryotubes at −80°C within 24 hours of collection.

#### Data management

Data are entered by the research nurses into a REDCap electronic data capture (EDC) tool with web-based interface,^52 53^ hosted by MSHR at Charles Darwin University. Laboratory data are entered by the laboratory scientist. The EDC tool includes built-in validation ranges to facilitate accurate data entry. An EDC audit trail maintains a record of entries and changes made. Logic and 10% data checks (EDC cross-checked with DCFs) are performed regularly. Error rates greater than 1% precipitate 100% checks of targeted data fields and further 10% checks of all data.

#### Statistical analyses

The RCT is being conducted and reported according to CONSORT (Consolidated Standards of Reporting Trials) guidelines.^54^ A detailed statistical analysis plan will be developed by the investigators and study statisticians prior to the unblinding and final analysis.

The primary ‘intention-to-treat’ analysis will compare the incidence of infant ARI (episodes/child/12 months) between active and placebo groups using a negative binomial regression model and producing an estimate of the incidence rate ratio (IRR) with 95%CIs. The model will include terms to account for repeat mothers and twins. There will be no imputation for missing data. Supplementation efficacy will be defined as (1−IRR)×100.

Secondary analyses (clinical) will include between group comparisons of (1) the proportion of children having any ARI episode <12 months (using a generalised linear model with effects presented as risk ratios), (2) time to first ARI presentation (using Cox regression with effects presented as HRs) and (3) the incidence of ARI subgroup outcomes (pneumonia, bronchiolitis and OM using negative binomial regression with effects presented as IRRs). Sensitivity analyses will include those (1) with hospitalised outcomes and (2) clinical outcomes requiring antibiotics. A per-protocol analysis (excluding those who missed >3 consecutive doses) will also be conducted for all clinical outcomes.

Secondary analyses (biological) will compare between-group differences in (1) circulating vitamin D levels: mean plasma 25OHD at each visit and breast milk 25OHD at birth (Student’s t-test), (2) microbiological outcomes: proportions of nasal swabs positive for *S. pneumoniae*, NTHi and RSV using χ^2^ tests and reporting risk ratios with 95% CIs and (3) immune function measures: median immune cell population counts in cord blood at birth and infant blood at 4 months; median circulating inflammatory marker levels in cord blood at birth and infant blood at 4 months; median CBMC population counts and CBMC challenge-induced cytokine levels (Wilcoxon rank-sum test); geometric mean concentrations of IgG and IgA to pertussis vaccine antigens in maternal plasma and breast milk; geometric mean concentrations of IgG and IgA PCV serotype antigens (five types) in infant saliva and plasma at age 4 months (Student’s t-test). All data will be analysed using Stata Statistical Software: Release V.17 (StataCorp) and GraphPad Prism V.9 (GraphPad Software, USA).

For all analyses, p values <0.05 will be considered statistically significant. No adjustments will be made for multiple comparisons.

### Monitoring

#### Data monitoring and harm assessment

An iDSMC was established prior to any participant recruitment. The committee includes experienced study independent statisticians, clinicians, endocrinologists and epidemiologists. SAEs (hospital admissions) and other AEs are monitored by study staff through hospital visits, participant contact and regular review of participant medical records. Each SAE is reported to an independent medical monitor (an experienced paediatrician not on the iDSMC) to review causality. SAEs deemed as potentially related to the study are reported to the iDSMC and Human Research Ethics Committee (HREC) within 72 hours. At regular intervals (<6 months), the iDSMC assess the totality of information relating to participant recruitment and retention, protocol deviations and violations, AEs and SAEs. Unblinding can be requested where concerns arise. With compelling evidence, the iDSMC can recommend cessation of the trial. Given vitamin D is low risk, there are no predefined stopping rules or planned interim analyses. A First Nations Reference Group oversees cultural aspects of the study.

## Discussion

Our ‘D-Kids’ RCT is the first to evaluate perinatal vitamin D supplementation against medically attended ARIs among Australian First Nations infants during the first year of life. The sample size of 314 infants is expected to provide 90% power to detect a 27.5% relative reduction in new ARI episodes between groups. Importantly, our mechanistic outcomes will further characterise the effects of vitamin D supplementation on infant immune function and respiratory microbiology.

This ‘D-Kids’ trial has several strengths. It addresses ARIs, an unmet health need, particularly among socioeconomically disadvantaged children. The study premise is guided by vitamin D and ARI data specific to the target population^3 24^ and the design incorporates key strengths of previous trials^12 27 31^ and guidelines^55^ including dose timing, concentration and frequency and potential effect size. Our study also addresses important knowledge gaps regarding the effectiveness among neonates and of practical weekly dosing regimens, and integrates immunological and microbiological outcomes. The weekly maternal (equivalent 2000 IU/day) and infant supplementation doses (equivalent 600 IU/day) were chosen to maintain vitamin D sufficiency (>50 nmol/L) throughout the active study period, taking into account previously published local infant vitamin D levels at birth.^24^ Importantly, there have been no SAEs associated with vitamin D supplementation in RCTs,^12 31^ as such, the balance of risk versus reward of a vitamin D strategy is likely to be highly favourable.

Of note, recent evidence suggests daily dosing is most effective clinically.^31^ Further, there is emerging biological evidence to suggest bolus dosing is ineffective due to upregulation of 24-hydroxylase (converts 25OHD and 1,25OH_2_D_3_ into less active 24-hydroxylated products) and FGF23 (inhibits 1α-hydroxylase which is necessary to locally convert circulating 25OHD into active 1,25OH_2_D_3_) that act to balance the vitamin D response.^56^ However, these studies^57 58^ compare large bolus doses >150 000 IU with daily doses and the biological effect of moderate weekly doses (<14 000) remains unclear. One meta-analysis suggests that daily dose equivalents of 2000 IU or less in individuals with circulating 25OHD<100 nmol/L are unlikely to induce the same down regulation in vitamin D function as large bolus doses.^59^ Our study will contribute valuable data on the clinical and immunological effects of weekly vitamin D dosing in infants.

There are also many challenges. The trial is being conducted throughout a pandemic. While the D-Kids trial has never officially paused, lockdowns, isolation and quarantine measures designed to reduce the spread of SARS-CoV-2 have restricted face to face contact, interaction with medical facilities and general travel, impacting participant recruitment and follow-up, and reducing the rates of ARI. As of December 2022, 184 infants had been recruited to the study. Several strategies have been implemented to mitigate slow recruitment including the addition of new study sites (notably Alice Springs in Central Australia), self-administered dosing, clinic assisted follow-up visits, and recruitment of twins and previously enrolled mothers. Notably, while self-administered dosing mimics the real-world setting, adherence (phone-based reporting) becomes more difficult to accurately monitor. With pandemic measures easing in 2023, we are optimistic about achieving our recruitment target.

Feasible interventions such as vitamin D supplementation show considerable potential against ARI but more evidence is required. Our study outcomes will make an important contribution to clinical practice, the medical literature and could have profound implications for disadvantaged populations where ARIs are common.

## Dissemination

### Registration

‘D-Kids’ is registered with the Australia and New Zealand Clinical Trial Registry: http://www.ANZCTR.org.au (ACTRN12618001174279). The current protocol version is 2.1 (last updated 16 September 2022).

### Protocol amendments

All protocol modifications are reported to the NT HREC for review and approval. Trial registries are regularly updated as required. Investigators, the iDSMB and other stakeholders, including participating community health services, are advised of important protocol amendments, such as those that may impact on participant safety, scientific validity, scope or ethical rigour. Substantive protocol amendments are agreed by the ‘D-Kids’ investigator team and are approved by HREC before implementation. Minor administrative amendments are documented in notes to file.

### Consent

Only appropriately trained staff conduct informed consent. Information is provided to the mother in written, verbal and pictorial formats, with language translation where requested. The study is explained to expectant mothers face to face, and they are provided with sufficient time to ask questions, discuss and consider participation of themselves and their child with relevant others and obtain further study details prior to signing and dating the informed consent form. The consent process includes explanations of all elements of consent according to GCP, the Declaration of Helsinki, National Health and Medical Research Council (NHMRC) requirements and according to local requests to ensure cultural safety (as recommended by the First Nations Child Health Reference Group.

Additional consent is sought from parents or guardians to use participant data and biological specimens for future research relating to child health respiratory studies. Options to refuse each or all requests are provided. Participants are also asked if they would like to be contacted about future research studies.

### Confidentiality

All identifiable information on study participants is retained in password-protected files and locked cabinets at study sites. Access to this information is only provided to authorised study staff, unless required by legislative or regulatory agencies and the HREC. No identifying information will be included in study reports. Clinical specimens are labelled with the participant randomisation number only and will be destroyed as per the NHMRC-based ethics statement.

### Declaration of interests

MSHR (NT, Australia) is the trial sponsor. Study investigators working at MSHR and partnering institutes are solely responsible for the design, conduct and reporting of this RCT. The investigators and protocol authors declare no conflicts of interest. The trial is funded by the NHMRC. The study medicine and placebo are manufactured and supplied free of charge by Ddrops, Ontario, Canada. Neither Ddrops or the NHMRC had/will have any role in the trial study design, conduct, analysis or reporting.

### Access to data

The final trial dataset will be under the custody of the trial sponsor, MSHR, NT, Australia. The principal investigator, study statistician and senior data manager at MSHR will have access to all study data. Third party access to the final anonymised dataset will require written requests to be approved by the HREC, iDSMC, study investigators and the director of MSHR.

### Ancillary and post-trial care

All participants have access to ancillary care from their usual healthcare provider (local community health centre). Trial participants will be insured and indemnified by the MSHR for their involvement in the study. Injury due to study procedures will be considered trial related.

### Dissemination policy

Trial results will be communicated in aggregate to participant families and their communities via written and oral presentations. Trial results will then be published in peer-reviewed international journals, presented at relevant national and international conferences, and reported to local policy-makers (eg, NT and Australian government, Therapeutic Goods Administration, First Nations Reference Group). Results will be disseminated regardless of the magnitude or direction of effect. There will be no publication restrictions. Applications for third party access to deidentified trial data will be considered by study investigators if appropriately justified and compliant with ethical and privacy policies.

## Data Availability

No data are available. This is a protocol paper, and therefore, there are no associated data.

## References

[1] McAllister DA, Liu L, Shi T, et al. Global, regional, and national estimates of pneumonia morbidity and mortality in children younger than 5 years between 2000 and 2015: a systematic analysis. Lancet Glob Health 2019;7:e47–57. 10.1016/S2214-109X(18)30408-X 30497986 PMC6293057

[2] Moore HC, Lehmann D, de Klerk N, et al. Reduction in disparity for pneumonia hospitalisations between Australian indigenous and non-indigenous children. J Epidemiol Community Health 2012;66:489–94. 10.1136/jech.2010.122762 21258115

[3] Binks MJ, Beissbarth J, Oguoma VM, et al. Acute lower respiratory infections in indigenous infants in Australia’s Northern territory across three eras of pneumococcal conjugate vaccine use (2006-15): a population-based cohort study. Lancet Child Adolesc Health 2020;4:425–34. 10.1016/S2352-4642(20)30090-0 32450122

[4] Binks MJ, Moberley SA, Balloch A, et al. Pneumum: impact from a randomised controlled trial of maternal 23-valent pneumococcal polysaccharide vaccination on middle ear disease amongst indigenous infants, Northern territory, Australia. Vaccine 2015;33:6579–87. 10.1016/j.vaccine.2015.10.101 26529076

[5] Leach AJ, Wigger C, Beissbarth J, et al. General health, Otitis media, nasopharyngeal carriage and middle ear microbiology in northern territory aboriginal children vaccinated during consecutive periods of 10-valent or 13-valent pneumococcal conjugate vaccines. Int J Pediatr Otorhinolaryngol 2016;86:224–32. 10.1016/j.ijporl.2016.05.011 27260611

[6] McCallum GB, Binks MJ. The epidemiology of chronic suppurative lung disease and bronchiectasis in children and adolescents. Front Pediatr 2017;5:27. 10.3389/fped.2017.00027 28265556 PMC5316980

[7] Chang AB, Masel JP, Boyce NC, et al. Non-CF bronchiectasis: clinical and HRCT evaluation. Pediatr Pulmonol 2003;35:477–83. 10.1002/ppul.10289 12746947

[8] O’Grady K-AF, Torzillo PJ, Chang AB. Hospitalisation of indigenous children in the Northern territory for lower respiratory illness in the first year of life. Med J Aust 2010;192:586–90. 10.5694/j.1326-5377.2010.tb03643.x 20477735

[9] Bleakley AS, Licciardi PV, Binks MJ. Vitamin D modulation of the innate immune response to paediatric respiratory pathogens associated with acute lower respiratory infections. Nutrients 2021;13:276. 10.3390/nu13010276 33478006 PMC7835957

[10] Mora JR, Iwata M, von Andrian UH. Vitamin effects on the immune system: vitamins A and D take centre stage. Nat Rev Immunol 2008;8:685–98. 10.1038/nri2378 19172691 PMC2906676

[11] Grant CC, Kaur S, Waymouth E, et al. Reduced primary care respiratory infection visits following pregnancy and infancy vitamin D supplementation: a randomised controlled trial. Acta Paediatr 2015;104:396–404. 10.1111/apa.12819 25283480

[12] Martineau AR, Jolliffe DA, Hooper RL, et al. Vitamin D supplementation to prevent acute respiratory tract infections: systematic review and meta-analysis of individual participant data. BMJ 2017;356:i6583. 10.1136/bmj.i6583 28202713 PMC5310969

[13] Hughes DA, Norton R. Vitamin D and respiratory health. Clin Exp Immunol 2009;158:20–5. 10.1111/j.1365-2249.2009.04001.x 19737226 PMC2759054

[14] Lauridsen AL, Vestergaard P, Hermann AP, et al. Plasma concentrations of 25-hydroxy-vitamin D and 1,25-dihydroxy-vitamin D are related to the phenotype of GC (vitamin D-binding protein): a cross-sectional study on 595 early postmenopausal women. Calcif Tissue Int 2005;77:15–22. 10.1007/s00223-004-0227-5 15868280

[15] White JH. Vitamin D deficiency and the pathogenesis of Crohn's disease. J Steroid Biochem Mol Biol 2018;175:23–8. 10.1016/j.jsbmb.2016.12.015 28025175

[16] Lips P. Vitamin D physiology. Prog Biophys Mol Biol 2006;92:4–8. 10.1016/j.pbiomolbio.2006.02.016 16563471

[17] Holick MF. Vitamin D deficiency. N Engl J Med 2007;357:266–81. 10.1056/NEJMra070553 17634462

[18] Hoe E, Nathanielsz J, Toh ZQ, et al. Anti-inflammatory effects of vitamin D on human immune cells in the context of bacterial infection. Nutrients 2016;8:806. 10.3390/nu8120806 27973447 PMC5188461

[19] Wayse V, Yousafzai A, Mogale K, et al. Association of subclinical vitamin D deficiency with severe acute lower respiratory infection in Indian children under 5 Y. Eur J Clin Nutr 2004;58:563–7. 10.1038/sj.ejcn.1601845 15042122

[20] Roth DE, Shah R, Black RE, et al. Vitamin D status and acute lower respiratory infection in early childhood in Sylhet, Bangladesh. Acta Paediatr 2010;99:389–93. 10.1111/j.1651-2227.2009.01594.x 19900174

[21] Karatekin G, Kaya A, Salihoğlu O, et al. Association of subclinical vitamin D deficiency in newborns with acute lower respiratory infection and their mothers. Eur J Clin Nutr 2009;63:473–7. 10.1038/sj.ejcn.1602960 18030309

[22] Belderbos ME, Houben ML, Wilbrink B, et al. Cord blood vitamin D deficiency is associated with respiratory syncytial virus bronchiolitis. Pediatrics 2011;127:e1513–20. 10.1542/peds.2010-3054 21555499

[23] Shin YH, Yu J, Kim KW, et al. Association between cord blood 25-hydroxyvitamin D concentrations and respiratory tract infections in the first 6 months of age in a Korean population: a birth cohort study (COCOA). Korean J Pediatr 2013;56:439–45. 10.3345/kjp.2013.56.10.439 24244212 PMC3827492

[24] Binks MJ, Smith-Vaughan HC, Marsh R, et al. Cord blood vitamin D and the risk of acute lower respiratory infection in indigenous infants in the Northern territory. Med J Aust 2016;204:238. 10.5694/mja15.00798 27031398

[25] Grant CC, Crane J, Mitchell EA, et al. Vitamin D supplementation during pregnancy and infancy reduces aeroallergen sensitization: a randomized controlled trial. Allergy 2016;71:1325–34. 10.1111/all.12909 27060679

[26] Roth DE, Morris SK, Zlotkin S, et al. Vitamin D supplementation in pregnancy and Lactation and infant growth. N Engl J Med 2018;379:535–46. 10.1056/NEJMoa1800927 30089075 PMC6004541

[27] Grant CC, Stewart AW, Scragg R, et al. Vitamin D during pregnancy and infancy and infant serum 25-hydroxyvitamin D concentration. Pediatrics 2014;133:e143–53. 10.1542/peds.2013-2602 24344104

[28] Thorne-Lyman A, Fawzi WW. Vitamin D during pregnancy and maternal, neonatal and infant health outcomes: a systematic review and meta-analysis. Paediatr Perinat Epidemiol 2012;26 Suppl 1:75–90. 10.1111/j.1365-3016.2012.01283.x 22742603 PMC3843348

[29] Hollis BW, Johnson D, Hulsey TC, et al. Vitamin D supplementation during pregnancy: double-blind, randomized clinical trial of safety and effectiveness. J Bone Miner Res 2011;26:2341–57. 10.1002/jbmr.463 21706518 PMC3183324

[30] Manaseki-Holland S, Maroof Z, Bruce J, et al. Effect on the incidence of pneumonia of vitamin D supplementation by quarterly bolus dose to infants in Kabul: a randomised controlled superiority trial. Lancet 2012;379:1419–27. 10.1016/S0140-6736(11)61650-4 22494826 PMC3348565

[31] Jolliffe DA, Camargo CA, Sluyter JD, et al. Vitamin D supplementation to prevent acute respiratory infections: a systematic review and meta-analysis of aggregate data from randomised controlled trials. Lancet Diabetes Endocrinol 2021;9:276–92. 10.1016/S2213-8587(21)00051-6 33798465

[32] Australian Bureau of Statistics. Population estimates of aboriginal and torres strait Islander Australians. Canberra, 2016. Available: https://www.abs.gov.au/ausstats/abs@.nsf/Lookup/3238.0.55.001main+features1June%202016 [accessed 12 Jul 2019].

[33] Valery PC, Morris PS, Byrnes CA, et al. Long-term azithromycin for indigenous children with non-cystic-fibrosis bronchiectasis or chronic suppurative lung disease (Bronchiectasis intervention study): a multicentre, double-blind, randomised controlled trial. Lancet Respir Med 2013;1:610–20. 10.1016/S2213-2600(13)70185-1 24461664

[34] Goyal V, Grimwood K, Byrnes CA, et al. Amoxicillin-clavulanate versus azithromycin for respiratory exacerbations in children with bronchiectasis (BEST-2): a multicentre, double-blind, non-inferiority, randomised controlled trial. Lancet 2018;392:1197–206. 10.1016/S0140-6736(18)31723-9 30241722 PMC7159066

[35] Chang AB, Toombs M, Chatfield MD, et al. Study protocol for preventing early-onset pneumonia in young children through maternal Immunisation: a multi-centre randomised controlled trial (Pneumatters). Front Pediatr 2021;9:781168. 10.3389/fped.2021.781168 35111703 PMC8802227

[36] Eyles DW, Morley R, Anderson C, et al. The utility of neonatal dried blood spots for the assessment of neonatal vitamin D status. Paediatr Perinat Epidemiol 2010;24:303–8. 10.1111/j.1365-3016.2010.01105.x 20415760

[37] Maunsell Z, Wright DJ, Rainbow SJ. Routine Isotope-dilution liquid chromatography-tandem mass spectrometry assay for simultaneous measurement of the 25-hydroxy metabolites of vitamins D2 and D3. Clin Chem 2005;51:1683–90. 10.1373/clinchem.2005.052936 16020493

[38] Binks MJ, Bleakley AS, Rathnayake G, et al. Can dried blood spots be used to accurately measure vitamin D metabolites? Clin Chim Acta 2021;518:70–7. 10.1016/j.cca.2021.03.003 33713691

[39] Satzke C, Turner P, Virolainen-Julkunen A, et al. Standard method for detecting upper respiratory carriage of Streptococcus pneumoniae: updated recommendations from the World Health Organization Pneumococcal carriage working group. Vaccine 2013;32:165–79. 10.1016/j.vaccine.2013.08.062 24331112

[40] Smith-Vaughan HC, Binks MJ, Beissbarth J, et al. Bacteria and viruses in the nasopharynx immediately prior to onset of acute lower respiratory infections in indigenous Australian children. Eur J Clin Microbiol Infect Dis 2018;37:1785–94. 10.1007/s10096-018-3314-7 29959609 PMC7088242

[41] Binks MJ, Cheng AC, Smith-Vaughan H, et al. Viral-bacterial co-infection in Australian indigenous children with acute Otitis media. BMC Infect Dis 2011;11:161. 10.1186/1471-2334-11-161 21649905 PMC3128050

[42] Pizzutto SJ, Yerkovich ST, Upham JW, et al. Children with chronic suppurative lung disease have a reduced capacity to synthesize interferon-gamma in vitro in response to non-Typeable haemophilus influenzae. PLoS One 2014;9:e104236. 10.1371/journal.pone.0104236 25111142 PMC4128648

[43] Anderson J, Thang CM, Thanh LQ, et al. Immune profiling of cord blood from preterm and term infants reveals distinct differences in pro-inflammatory responses. Front Immunol 2021;12:777927. 10.3389/fimmu.2021.777927 34790206 PMC8591285

[44] Park C, Huh DH, Han SB, et al. Development and implementation of standardized method for detecting immunogenicity of acellular pertussis vaccines in Korea. Clin Exp Vaccine Res 2019;8:35–42. 10.7774/cevr.2019.8.1.35 30775349 PMC6369126

[45] Balloch A, Licciardi PV, Leach A, et al. Results from an inter-laboratory comparison of pneumococcal serotype-specific IgG measurement and critical parameters that affect assay performance. Vaccine 2010;28:1333–40. 10.1016/j.vaccine.2009.11.011 19932671

[46] Wang TJ, Zhang F, Richards JB, et al. Common genetic determinants of vitamin D insufficiency: a genome-wide association study. Lancet 2010;376:180–8. 10.1016/S0140-6736(10)60588-0 20541252 PMC3086761

[47] Hoggard M, Jacob B, Wheeler D, et al. Multiomic analysis identifies natural intrapatient temporal variability and changes in response to systemic corticosteroid therapy in chronic rhinosinusitis. Immun Inflamm Dis 2021;9:90–107. 10.1002/iid3.349 33220024 PMC7860613

[48] Kia E, Wagner Mackenzie B, Middleton D, et al. Integrity of the human faecal microbiota following long-term sample storage. PLoS One 2016;11:e0163666. 10.1371/journal.pone.0163666 27701448 PMC5049846

[49] Kearns T, Clucas D, Connors C, et al. Clinic attendances during the first 12 months of life for aboriginal children in five remote communities of northern Australia. PLoS ONE 2013;8:e58231. 10.1371/journal.pone.0058231 23469270 PMC3585931

[50] Keene ON, Jones MRK, Lane PW, et al. Analysis of exacerbation rates in asthma and chronic obstructive pulmonary disease: example from the TRISTAN study. Pharm Stat 2007;6:89–97. 10.1002/pst.250 17230434

[51] Leach AJ, Wilson N, Arrowsmith B, et al. Immunogenicity, Otitis media, hearing impairment, and nasopharyngeal carriage 6-months after 13-valent or ten-valent booster pneumococcal conjugate vaccines, stratified by mixed priming schedules: Previx_Combo and Previx_Boost randomised controlled trials. Lancet Infect Dis 2022;22:1374–87. 10.1016/S1473-3099(22)00272-9 35772449

[52] Harris PA, Taylor R, Minor BL, et al. The Redcap consortium: building an international community of software platform partners. J Biomed Inform 2019;95:103208. 10.1016/j.jbi.2019.103208 31078660 PMC7254481

[53] Harris PA, Taylor R, Thielke R, et al. Research electronic data capture (Redcap)--A metadata-driven methodology and workflow process for providing translational research Informatics support. J Biomed Inform 2009;42:377–81. 10.1016/j.jbi.2008.08.010 18929686 PMC2700030

[54] Schulz KF, Altman DG, Moher D, et al. Statement: updated guidelines for reporting parallel group randomised trials. Trials 2010;11:32. 10.1186/1745-6215-11-32 21350618 PMC3043330

[55] Nowson CA, McGrath JJ, Ebeling PR, et al. Vitamin D and health in adults in Australia and New Zealand: a position statement. Med J Aust 2012;196:686–7. 10.5694/mja11.10301 22708765

[56] Griffin G, Hewison M, Hopkin J, et al. Perspective: vitamin D supplementation prevents Rickets and acute respiratory infections when given as daily maintenance but not as intermittent bolus: implications for COVID-19. Clin Med 2021;21:e144–9. 10.7861/clinmed.2021-0035 PMC8002781 33593830

[57] Turner C, Dalton N, Inaoui R, et al. Effect of a 300 000-IU loading dose of ergocalciferol (vitamin D2) on circulating 1,25(OH)2-vitamin D and fibroblast growth factor-23 (FGF-23) in vitamin D insufficiency. J Clin Endocrinol Metab 2013;98:550–6. 10.1210/jc.2012-2790 23284004

[58] Ketha H, Thacher TD, Oberhelman SS, et al. Comparison of the effect of daily versus bolus dose maternal vitamin D(3) supplementation on the 24,25-dihydroxyvitamin D(3) to 25-hydroxyvitamin D(3) ratio. Bone 2018;110:321–5. 10.1016/j.bone.2018.02.024 29486367 PMC5878742

[59] Zittermann A, Berthold HK, Pilz S. The effect of vitamin D on fibroblast growth factor 23: a systematic review and meta-analysis of randomized controlled trials. Eur J Clin Nutr 2021;75:980–7. 10.1038/s41430-020-00725-0 32855522 PMC8510890

