# Supplementary material for: Randomised controlled trial of perinatal vitamin D supplementation to prevent early-onset acute respiratory infections among Australian First Nations children: the ‘D-Kids’ study protocol
Source: BMJ Open Respir Res. 2023 Aug 16;10(1):e001646. doi: 10.1136/bmjresp-2023-001646 (PMC10432658; doi:10.1136/bmjresp-2023-001646)
Supplement: Supplementary data [file bmjresp-2023-001646supp001.pdf]

menzies school of health research

Supplementary Material 1: Clinical outcome algorithm (links to 'collected data' on page 2)

PRIMARY OUTCOME:

1. **Acute respiratory infection (ARI)**
   - Any documented ARI diagnosis (**1ABCD)**

SECONDARY OUTCOMES:

2. **Acute LOWER respiratory infection (ALRI)** defined by:
   - A documented ALRI diagnosis (**1A**)
   - Any ARI diagnosis (**1BCD**) where the following are present:
     - ➢ Low oxygen saturation OR Increased respiratory rate for age (**2A**) OR Acute cough (**2C**) OR Increased work of breathing (WOB, **3A**) OR Abnormal auscultation signs (**3B**) OR Abnormal chest X-ray (**4A**) OR Wheeze **(from 3B)**

   ALRI Subcategories

   3. **Pneumonia** defined by:
      - A documented pneumonia diagnosis (**from 1A**)
      - A documented ARI diagnosis (**1ABC**) AND any of the following:
        - ➢ Acute cough (**2C**) AND Increased WOB (**3A**)
        - ➢ [Acute cough (**2C**) OR Increased WOB (**3A**)] AND [Fever (**2B**) OR Low oxygen saturation OR Increased respiratory rate for age (**2A**) OR Abnormal auscultation signs (**3B**) OR General symptoms of being unwell (**5**)]
        - ➢ Blood culture positive for *S. pneumoniae* or *H. influenzae* (**from 5B**)
        - ➢ Abnormal chest X-ray (**4A**)

   4. **Bronchiolitis** defined by:
      - A documented bronchiolitis diagnosis
      - Any documented ALRI diagnosis (**1ABC**) AND any of the following:
        - ➢ Wheeze (**from 3B**) OR Detection of RSV (**from 5A**)

5. **Acute UPPER respiratory infection (AURI)** defined by:
   - A documented AURI (**1B**) or other ARI diagnosis (**1C**) not classified as an ALRI or OM above.

6. **Otitis Media (OM)** defined by:
   - A documented OM diagnosis (**1D**)

Sensitivity analyses: *(a) Hospital only (b) Antibiotics prescribed*

Binks MJ, *et al. BMJ Open Respir Res* 2023; 10:e001646. doi: 10.1136/bmjresp-2023-001646

menzies school of health research

COLLECTED DATA

1. Documented diagnosis in medical record

A. ALRI	*B. AURI*	*C. Non-Specific*	*D. Otitis media (OM)*
Bronchiolitis<br>Bronchitis<br>Pneumonia<br>Influenza Like Illness<br>Whooping cough<br>LRTI/ALRI<br>Chest infection<br>Croup	Tonsillitis<br>Throat infection<br>URTI	Other RTI<br>Cough	OME<br>AOM<br>Non-specific OM

ALRI: acute lower respiratory infection. **LRTI:** lower respiratory tract infection. **URTI:** upper respiratory tract infection. **OME**: otitis media with effusion. **AOM**: acute otitis media.

2. Primary signs and symptoms

A. Respiratory vitals	*B. Fever*	*C. Acute Cough<14d*
O_2<92%<br>RR>50 (bpm)	Temperature>37.5°C	Wet cough<br>Dry cough

RR: respiratory rate. **bpm:** breaths per minute.

3. Secondary signs and symptoms

A. Work of Breathing	*B. Auscultation*	*C. Nasopharynx*	*D. Throat*	*E. General*
Dyspnoea<br>Chest indrawing<br>Rib recession<br>Tracheal tug<br>Nasal flaring<br>Grunting	Wheeze<br>Crackles/Crepitations/Riles<br>Stridor<br>AE equal	Nasal Discharge	Red<br>Pus<br>Enlarged tonsils	Unable to sit or feed<br>Vomiting

4. Radiographic signs and symptoms

A. X-ray
Patchy/consolidated

5. Pathogen identified

A. Respiratory swab	*B. Blood Culture*
Influenza A/B<br>RSV<br>SARS-COV2<br>Pertussis<br>*S. pneumoniae*<br>*H. influenzae*<br>Rhinovirus<br>Adenovirus<br>Enterovirus<br>Parainfluenza virus<br>Coronavirus<br>Other	*S. pneumoniae*<br>*H. influenzae*<br>*S. aureus*<br>*S. pyogenes*<br>*E. coli*<br>*N. meningitidis*<br>*K. pneumoniae*<br>*Pseudomonas species*<br>*Enterobacter species*<br>*Other Streptococcal species*

RSV: respiratory syncytial virus. **SARS-CoV-2**: severe acute respiratory syndrome coronavirus 2.

6. Inflammatory markers

**Cellularity**
Neutrophil/White Cell Count>15%

7. Treatment

A. Antibacterial	*B. Antiviral*	*C. Anti-inflammatory*	*D. Bronchodilator*	*E. Airway*
Antibiotics<14d	Antivirals	NSAID<br>SAID	Salbutamol	Oxygen

NSAID: non-steroidal anti-inflammatory drug. **SAID**: steroidal anti-inflammatory drug

Binks MJ, *et al. BMJ Open Respir Res* 2023; 10:e001646. doi: 10.1136/bmjresp-2023-001646
